# The role of Polish school nurses in the oral health promotion for 7–19 year-old children and adolescents

**DOI:** 10.1007/s40368-020-00546-6

**Published:** 2020-06-24

**Authors:** J. Baginska, E. Rodakowska, A. Kobus, A. Kierklo

**Affiliations:** 1 Department of Dentistry Propaedeutics, Medical University of Bialystok, ul. Szpitalna 30, 15-295 Bialystok, Poland; 2 Depertment of Restorative Dentistry, Medical University of Bialystok, Bialystok, Poland; 3 Present Address: Department of Clinical Dentistry-Cariology, University of Bergen, Bergen, Norway

**Keywords:** Dental health education, School nurse, School-based preventive programs, Caries

## Abstract

**Purpose:**

The assessment of the role of school nurses in the oral health education and counselling of children and adolescents aged 7–19 years.

**Methods:**

A self-administered questionnaire was used to evaluate nurses’ practice in oral health education, previous training in caries prevention, collaboration with a dentist and self-assessment of knowledge. Data were analysed with the Chi square test.

**Results:**

The study group consisted of 140 Polish school nurses. Respondents declared the following activities: dietary counselling (99.2%), oral hygiene education (92.8%), NHS-funded supervised fluoride prophylaxis (82.8%), and caries screenings (4.3%). 47.1% participated in training on caries prevention, 25.7% had a collaboration with a dentist. Nurses from schools located in towns less frequently provided oral hygiene education (*p* < 0.005) and dietary counselling, but more often had a collaboration with a dentist (*p* < 0.05). The youngest nurses were more confident about their knowledge (*p* < 0.05). There was an association between participation in training on caries prevention and positive opinion on a school-based fluoride prophylaxis (*p* < 0.01). Nurses who did not include dental topics in their practice worked mainly with pupils older than 15 years, had shorter experience (*p* < 0.05), secondary education, worked in urban areas and had no training on dental problems (*p* < 0.01).

**Conclusion:**

Polish school nurses have potentially crucial roles in improving the oral health status in pupils through oral hygiene education, dietary counselling and fluoride prophylaxis included in their duties.

## Introduction

Untreated dental caries is the most widespread disease in the world, affecting in 2010 about 35% of the global population, and the fourth most expensive chronic disease to treat (Kassebaum et al. 2007) Caries is considered to be a complex, multifactorial, lifestyle-dependent condition. Improper health-related behaviours as frequent consumption of fermentable carbohydrates, neglecting daily oral hygiene practices and avoiding dental appointments play a crucial role in the caries development. It has been proven that even a low reduction of sugar intake can improve epidemiological situation of caries (Olczak-Kowalczyk et al. 2016). The current United Nations (UN) policy classes oral problems among a group of non-communicable diseases (NCDs) based on the same risk factors as other chronic conditions (United Nations 2017). The common risk approach provides a rationale for integrating oral health promotion into general health messages (Benzian et al. 2017).

Oral hygiene habits and dietary preferences develop during childhood and adolescence, and after that period it is difficult to change improper behaviours. Raising the level of dental caries prevention awareness constitutes one of key elements in the healthy lifestyle promotion. It is possible through health education for children and adolescents. Schools are, apart from home, places where healthy behaviours should be introduced and developed (Macnab 2015). School-based child oral hygiene programmes may contribute to improving oral health (Lai et al. 2016; Geetha Priya et al. 2019). The impact of school on children’s dental health is greater in low-income families (Navarro 2010). Activities for promoting oral health in schools have effect, through children and adolescents, also on their families and local communities (Benzian et al. 2015). There are three main topic areas within the oral health education including: oral hygiene, proper nutrition and using fluoride compounds. The class topics should be adapted to the age of pupils and varied using models, slides, videos, games, computer programmes, role-playing and other activities (Kwan 2005; Ahire et al. 2012; Geetha Priya et al. 2019).

Non-oral health professions may contribute to the prevention and control of dental diseases (Benzian et al. 2015). Every health provider should have basic competence in promoting oral health and in recognizing an oral disease so that he or she may reduce common risk factors for oral diseases and improve overall health in individuals and communities. According to the latest WHO report, health professionals should be globally competent and locally relevant (WHO 2013). In a Competency Matrix for Global Oral Health, nurses were included in the third category of competence together with physicians, physician assistants and pharmacists (Benzian et al. 2015). Especially school nurses can contribute to the oral health of pupils (Naseri-Salahshour et al. 2010; Tetuan 2004; Buerlein 2010). They should have competences to provide general oral hygiene and nutrition education, to make caries risk assessments, to apply fluoride varnish or gels, and to give first aid in dental emergencies like pain, swelling or trauma. After short training, nurses can perform extra- and intraoral screening for dental caries and other oral conditions (Tetuan et al. 2005). Nurses can play an important role as oral health care providers and educators in the special needs school setting (Oliva 2013). They should be partners in oral health counselling together with dentist and family doctors.

Despite the importance of school nurses in oral health promotion, little is known about their practices. The purpose of this survey was to assess the role of school nurses in Poland in oral health education and counselling.

## Methods

A cross-sectional study with the use of a self-administered questionnaire was conducted between January and April 2014 on randomly selected school nurses working in Polish provinces. The protocol of the study was approved by the Bioethical Committee of the Medical University of Bialystok, Poland.

### Study population

The following assumptions were taken to establish the minimum sample size: the number of school nurses based on data from the Polish Ministry of Health of about 8000, the percentage of nurses who include oral health in their duties (estimated fraction size) based on a pilot study of 90%, the confidence level of 95%, maximum error of 5%. The sample size was estimated to be at least 136 nurses. Due to the anticipated low response rate known from other studies at the level of about 40–50%, 333 school nurses were randomly selected from the internet database of the Central Statistical Office of Poland (Central Statistical Office 2014). They were sent a questionnaire together with a pre-paid return envelope, the information about the purpose of the study and a form of consent to the participation in the survey.

### Questionnaire

A structured self-administered questionnaire was previously used for testing purposes in a pilot study on 37 school nurses. The first part of the questionnaire consisted of demographic questions regarding the period of work experience as a school nurse, the nurse’s education level and the kind of their school’s location. The second part included a self-assessment of knowledge regarding dental caries, previous training in caries prevention, kind and frequency of actions taken at schools, and collaboration with a dentist.

### Statistical analysis

Data were analysed with Statistica 10.0. The Chi square with the Yates correction, if needed, was adopted. The level of statistical significance was *p* < 0.05.

## Results

Only164 nurses responded to the survey, which gave a response rate at the level of 49.25%; fifteen questionnaires were excluded due to incomplete data. Nine nurses declared that they did not provide any kind of oral health education to pupils. The final study group consisted of 140 school nurses. Table 1 presents the demographic data of the study group versus those nurses who did not include caries prevention in their practice. Most nurses from the study group had long experience as a school nurse – 62 of them worked longer than 25 years and 47 worked between 16 and 25 years; the average work experience was 23 ± 9.1. They mainly had secondary education; only 39 (27.8%) persons had a university degree. The location of nurses’ working place was as follows: a city – 40 (28.6%), a town – 63 (45%) and a rural area – 37 (26.4%). The mean number of pupils per one school nurse was 807.7 ± 258.3. Most respondents worked in more than one school (mean number 2.9 ± 1.6). One hundred and twenty six nurses worked in primary schools (pupils aged 7–12), ninety two worked in junior high schools (pupils aged 13–15), and eighty worked in high schools (pupils aged 16–19).

**Table 1:** Demographic differences between nurses who include and do not include oral health education in their practice (*χ*² test, *p* < 0.05, * Yates correction)

	Nurses including oral health education in their practice (*N* = 140)	Nurses not including oral health education in their practice (*N* = 9)	*p *value
Work experience (years)			0.047
1–15	31 (22.14%)	5 (55.69%)	
16–25	47 (33.57%)	3 (33.33%)	
26 and more	62 (44.29%)	1 (11.11%)	
Education			NS*
Secondary	101 (72.14%)	9 (100%)	
University	39 (27.86%)	0	
Location of school			NS
Rural area	37 (26.43%)	0	
Urban area	63 (45%)	6 (66.67%)	
City	40 (28.57%)	3 (33.33%)	
Training on caries prevention			0.008
Yes	74 (52.86%)	0	
No	66 (47.14%)	9 (100%)	

Taking into account the respondents who did not include dental topics in their practice, they had statistically significantly shorter experience as school nurses (*p* < 0.05), all of them had secondary education, worked in urban areas and no one participated in any training on dental problems (*p* < 0.01). They worked mainly in high schools (9 nurses) and junior high schools (5 nurses). Only one respondent worked in a primary school. The mean number of workplaces was 1.8 ± 0.6.

Tables 2 and 3 present participants’ responses to the questions asked in the questionnaire. Surveyed nurses declared the following activities: dietary counselling (99.2%), oral hygiene education (92.8%), NHS-funded supervised fluoride prophylaxis (82.8%), and caries screenings (4.3%). Three quarters of respondents declared that pupils often asked them for advice or help in dental problems, but only for 46.4% of nurses the dental issues were an important aspect in their practice. Less than a half of the study group (47.1%) participated in any training on caries prevention and only one quarter (25.7%) had a collaboration with a dentist.

**Table 2:** The distribution of answers to the questions according to school nurses’ level of education and work experience as a school nurse (*χ*² test, *p* < 0.05, * Yates correction, *p* < 0.05, NS—not significant)

	Total N (%)	Education *N* (%)			Work experience (years) N (%)			
		Secondary	University	*p* value	1–15	16–25	> 25	*p* value
How often do you give an oral hygiene instruction in each class?				NS				NS
At least once a year	121 (86.4%)	88 (87.2%)	33 (84.6%)		29 (93.6%)	43 (91.5%)	49 (79.0%)	
Occasionally (on demand)	9 (6.4%)	7 (6.9%)	3 (7.7%)		1 (3.2%)	3 (6.4%)	6 (9.7%)	
Never	10 (7.2%)	6 (5.9%)	3 (7.7%)		1 (3.2%)	1 (2.1%)	7 (11.3%)	
Do you perform NHS-funded fluoride prophylaxis?				NS*				NS
Yes	116 (82.8%)	83 (82.2%)	33 (84.6%)		29 (93.5%)	38 (80.8%)	49 (79.0%)	
No	24 (17.2%)	18 (17.2%)	6 (15.4%)		2 (6.5%)	9 (19.2%)	13 (21.0%)	
How often do you hold discussions on the role of diet in caries prevention?				NS				NS
At least once a year	114 (81.4%)	80 (79.2%)	34 (87.2%)		28 (90.3%)	38 (80.9%)	48 (77.4%)	
Occasionally (on demand)	25 (17.8%)	20 (19.8%)	5 (12.8%)		3 (9.7%)	8 (1.7%)	14 (22.6%)	
Never	1 (0.8%)	1 (1.0%)	0		0	1 (2.1%)	0	
Do you perform screening for dental caries?				NS*				NS
Yes	6 (4.3%)	4 (3.9%)	3 (5.1%)		1 (3.2%)	2 (4.2%)	3 (4.8%)	
No	134 (95.7%)	97 (96.0%)	37 (94.9%)		30 (96.8%)	45 (5.8%)	59 (95.2%)	
Are oral health issues an important part of your work?				NS				NS
Yes	65 (46.4%)	44 (43.6%)	21 (53.8%)		20 (64.5%)	21 (44.7%)	24 (38.7%)	
No	68 (48.6%)	51 (50.5%)	17 (43.6%)		10 (32.3%)	22 (46.8%)	36 (58.1%)	
No opinion	7 (5.0%)	6 (5.9%)	1 (2.6%)		1 (3.2%)	4 (8.5%)	2 (3.2%)	
Did you participate in training on dental caries prevention?				NS				NS
Yes	66 (47.1%)	46 (45.5%)	20 (51.3%)		14 (45.1%)	21 (44.7%)	31 (50.0%)	
No	74 (52.9%)	55 (54.4%)	19 (48.7%)		17 (54.9%)	26 (55.3%)	31 (50.0%)	
Do you collaborate with a dentist?				NS*				NS
Yes	36 (25.7%)	23 (22.8%)	13 (33.3%)		7 (22.6%)	9 (19.1%)	20 (32.3%)	
No	104 (74.3%)	76 (77.2%)	26 (66.7%)		24 (77.4%)	38 (80.9%)	42 (67.7%)	
Is there a dental office in your school?				NS				NS
Yes	30 (21.4%)	19 (18.8%)	11 (28.2%)		4 (12.9%)	13 (27.7%)	13 (21.0%)	
No	101 (72.2%)	76 (75.3%)	25 (64.1%)		26 (83.9%)	31 (65.9%)	44 (70.9%)	
In some schools	9 (6.4%)	6 (5.9%)	3 (7.7%)		1 (3.2%)	3 (6.4%)	5 (8.1%)	
Do pupils with dental problems come to see you?				NS				NS
Often	105 (75.0%)	74 (73.3%)	31 (79.5%)		25 (80.6%)	36 (76.6%)	44 (71.0%)	
Occasionally	29 (20.7%)	22 (21.8%)	7 (17.9%)		6 (19.4%)	8 (17.0%)	15 (24.2%)	
No	6 (4.3%)	5 (4.9%)	1 (2.6%)		0	3 (6.4%)	3 (4.8%)	
How do you assess your knowledge of caries prevention?				NS*				0.022
Adequate	111 (79.3%)	81 (80.2%)	30 (76.9%)		30 (96.8%)	34 (72.3%)	47 (75.8%)	
Not adequate	29 (20.7%)	20 (19.8%)	9 (23.1%)		1 (3.2%)	13 (27.7%)	15 (24.2%)	
What is your opinion about school-based fluoride prophylaxis?				NS				0.042
Very efficient	35 (25.0%)	25 (24.7%)	10 (25.6%)		11 (35.5%)	12 (25.5%)	12 (19.3%)	
Efficient	75 (53.6%)	57 (56.4%)	18 (46.1%)		19 (61.3%)	27 (57.5%)	29 (46.8%)	
Not efficient	24 (17.2%)	4 (4.0%)	2 (5.1%)		0	2 (4.2%)	4 (6.4%)	
No opinion	24 (17.2%)	15 (14.8%)	9 (23.1%)		1 (3.2%)	6 (12.8%)	17 (27.4%)

**Table 3:** The distribution of answers to the questions according to the location of school and training on caries prevention methods (*χ*² test, *p* < 0.05, *Yates correction, *p* < 0.05, NS—not significant)

	Location of school N (%)				Training on caries prevention N (%)		
	Rural area	Town	City	*p* value	Yes	No	*p* value
How often do you give an oral hygiene instruction in each class?				0.003			NS
At least once a year	36 (97.3%)	47 (74.6%)	38 (95.0%)		65 (87.8%)	56 (84.9%)	
Occasionally (on demand)	1 (2.7%)	7 (11.1%)	2 (5.0%)		7 (9.5%)	3 (4.5%)	
Never	0	9 (14.3%)	0		2 (2.7%)	7 (10.6%)	
Do you perform NHS-funded fluoride prophylaxis?				0.019			NS
Yes	34 (91.9%)	46 (73.0%)	36 (90.0%)		64 (86.5%)	52 (78.8%)	
No	3 (8.1%)	17 (27.0%)	4 (10%)		10 (13.5%)	54 (21.2%)	
How often do you hold discussions on the role of diet in caries prevention?				NS			NS
At least once a year	31 (83.8%)	49 (77.8%)	34 (85.0%)		42 (83.8%)	52 (78.8%)	
Occasionally (on demand)	6 (16.2%)	13 (20.6%)	6 (15%)		12 (16.2%)	13 (19.7%)	
Never	0	1 (1.6%)	0		00	1 (1.5%)	
Do you perform screening for dental caries?				NS			NS
Yes	1 (2.7%)	5 (7.9%)	0		4 (5.4%)	2 (3.0%)	
No	36 (97.3%)	58 (92.1%)	40 (100%)		70 (94.6%)	64 (97.0%)	
Are oral health issues an important part of your work?				NS			NS
Yes	21 (56.8%)	25 (39.7%)	19 (47.5%)		38 (51.4%)	27 (40.9%)	
No	16 (42.2%)	32 (50.8%)	20 (50%)		33 (44.6%)	35 (53.0%)	
No opinion	0	6 (9.5%)	1 (2.5%)		3 (4%)	4 (6.1%)	
Did you participate in training on dental caries prevention?				NS			
Yes	18 (48.6%)	27 (42.9%)	21 (52.5%)		–	–	–
No	9 (51.4%)	36 (57.1%)	19 (47.5%)		–	–	–
Do you collaborate with a dentist?				0.039			NS
Yes	9 (24.3%)	22 (34.9%)	5 (12.5%)		16 (21.6%)	20 (30.3%)	
No	28 (75.7%)	41 (65.1%)	35 (87.5%)		58 (78.4%)	46 (69.7%)	
Is there a dental office in your school?				0.043			NS
Yes	6 (16.2%)	18 (22.6%)	6 (15.0%)		11 (14.9%)	19 (28.8%)	
No	29 (78.4%)	98 (60.3%)	34 (85.0%)		58 (78.4%)	43 (65.1%)	
In some schools	2 (5.4%)	7 (11.1%)	0		5 (6.7%)	4 (6.1%)	
Do pupils with dental problems come to see you?				NS			0.039
Often	23 (62.2%)	47 (74.6%)	35 (87.5%)		62 (83.8%)	43 (65.2%)	
Occasionally	11 (29.7%)	14 (22.2%)	4 (10%)		10 (13.5%)	19 (28.8%)	
No	3 (8.1%)	2 (3.2%)	1 (2.5%)		2 (2.7%)	4 (6.1%)	
How do you assess your knowledge of caries prevention?				NS			NS
Adequate	25 (67.6%)	53 (84.1%)	33 (82.5%)		60 (81.1%)	51 (77.3%)	
Not adequate	12 (32.4%)	10 (15.9%)	7 (17.5%)		14 (18.9%)	15 (22.7%)	
What is your opinion about school-based fluoride prophylaxis?				NS			0.009
Very efficient	9 (24.3%)	21 (33.3%)	5 (12.5%)		11 (16.6%)	24 (33.4%)	
Efficient	22 (59.5%)	28 (44.4%)	25 (62.5%)		44 (66.7%)	31 (41.9%)	
Not efficient	1 (2.7%)	2 (3.2%)	3 (7.5%)		4 (6.1%)	2 (2.7%)	
No opinion	5 (13.5%)	12 (19.1%)	7 (17.5%)		7 (10.6%)	17 (22.9%)	

With regard to oral hygiene education and dietary counselling, there were no significant differences depending on the nurse’s education level and training in caries prevention. It was similar for the work experience, however, the oldest group least frequently provided any oral health education. Nurses working at schools located in towns less frequently than others provided oral hygiene education (*p* < 0.005) and dietary counselling (non-significant). On the other hand, they more often declared to have a collaboration with a dentist (*p* < 0.05) and more often reported that there was a dental office in their school (*p* < 0.05).

Respondents felt confident about their knowledge of caries pervention methods, only one fifth (20.7%) found it to be insufficient. The youngest group was statistically significantly more confident that older nurses (*p* < 0.05), and nurses from rural areas were less condifident than others, however, this observation was not supported by a statistical analysis. Both nurses who participated and those who did not participate in training on caries prevention were similarly confident about their knowledge. There was an association bewteen the participation in training on caries prevention and the nurses’ declaration that pupils often asked them for adivice on dental issues (*p* < 0.05) and their positive opinion on a school-based fluoride prophylaxis (*p* < 0.01). Another factor influencing the nurses’ opinion on this programme was the work experience—the youngest group statistically significantly often found it to be efficient or very efficient (*p* < 0.05).

## Discussion

The sugar consumption in Poland exceeds the level recommended by the WHO by two times and has a strong correlation with the dental condition in adolescents (Olczak-Kowlaczyk et al. 2016). A positive trend in dental hygiene habits in Polish 12-year-old has been observed since the last decades of the twentieth century. The most significant changes were the increase of rural children cleaning teeth twice a day from 34.2 in 1978 to 54.8% in 2010 and flossing teeth every day from 1.5 to 20.9%, respectively (Gaszyńska et al. 2014). Despite these findings, the overall level of dental caries remains a major oral health problem in Poland affecting more than 90% of schoolchildren with the experience of mean dmft (decayed/missed/filled index for primary teeth) in 6-year-old at 5.3 and mean DMFT (Decayed/Missed/Filled index for permanent teeth) in 12-year-old at 2.81 and in 15-year-old at 5.75 (Olczak-Kowalczyk et al. 2019; Polish Ministry of Health 2020). Therefore, there is an urgent need for continuous education and oral health promotion in children and adolescents.

The healthcare provided to pupils in Polish schools belongs to guaranteed services and is subject to a Regulation of the Ministry of Health (2013). In the Regulation applicable at the time of the survey, the oral health education to be provided to pupils was mentioned as one of nurse duties. Taking such measures was declared by 85% of school nurses who responded to the invitation to participate in the study. 90% of respondents who did not educate pupils on oral health worked in high schools with adolescents older than 15 years, however, some of them had also younger pupils under their care.

The most frequent measures were dietary counselling and oral hygiene instructions. The fact that these activities were performed at least once a year in every class is to be assessed as positive because educating children and adolescents on a rational model of nutrition and a proper oral hygiene undoubtedly have a favourable influence on the oral status. The school is a right place where such education should be provided because it allows starting oral health promotion activities in early years and continuing them throughout the school education period. Halonen et al. (2013) evaluated a community-based oral health promotion on primary schoolchildren’s oral hygiene habits. They concluded that younger children strongly benefited from the oral health intervention, but older pupils, especially boys, needed a continuous health promotion. Lai et al. (2016) found that a one-semester educational programme on brushing and flossing conducted by school nurses in 10–11-olds was sufficient to establish long-term positive effects on oral condition.

A high percentage of pupils asking for advice or help in dental problems proved the role of school nurse as an oral health educator. The survey showed that nurses who participated in the training on caries prevention more frequently gave such consultations. It is highly probable that pupils considered them to be competent in this field. On the other hand, this survey showed that school nurses did not prioritize oral health topics. Less than a half of the subjects considered oral issues to be an important part of their work. This may explain the low response rate obtained in the present study—less than 50%. It is also noteworthy that in their study on the activities of Polish school nurses Oblacińska et al. (2017) took only the fluoride prophylaxis into account in their survey and did not include any questions concerning oral health education and counselling. In general, Polish school nurses had difficulties with obtaining materials to be used for preparing and holding health education classes for pupils, and did not receive the relevant support from the head teacher or from other teachers but a gap existing in this field is not only a Polish problem (Oblacińska et al. 2017; Kent and Clark 2018). A limited availability of toothbrush, toothpaste and education materials was reported as a main problem for establishing a school-based oral prevention programme (Kwan 2005). For public health nurses in Norway, one of the barriers for including oral health promotion in their routine was the lack of time (Skeie et al. 2011). A significant percentage of Polish school nurses work in more than one school because there are imposed minimal limits of pupils under the care of a single school nurse. Some of them spend only one or two days in a particular school, so their time for the oral health education is limited.

The majority of respondents (82.8%) conducted the NHS-funded fluoride prophylaxis in the form of supervised tooth brushing with a high-concentration fluoride gel six times during a school year. The programme is restricted to primary school children and the child’s participation depends on the parents’ informed consent and the presence/absence of the child at school. This prophylaxis is not only beneficial for children but is also an additional source of earning for a school nurse. Nurses working at schools located in towns less frequently declared such action; this finding could be explained by the presence of a dental office at schools located in towns, which might prompt dentists to conduct the fluoride prophylaxis in pupils. A positive finding is that respondents generally favourably assessed the NHS-funded fluoride prophylaxis. Especially the youngest group considered it to be efficient, in contrast to one quarter of respondents from the oldest group having no opinion or even a negative attitude. Due to the misinformation widespread by antifluoridation community activists, there are parents having doubts about the side effects (Ota et al. 2013; Hendaus et al. 2016). They may ask a school nurse for counselling whether to give their consent to the child’s participation in the NHS-funded programme, therefore it is very important for school nurses to be confident about the safety and the effectiveness of topical fluoridation. This survey showed that the lack of training on caries prevention resulted in an uncertain attitude to the school-based fluoride caries prevention. A previously published study showed some gaps in the Polish school nurses’ knowledge on the safety of high-concentration fluoride products, which clearly indicate that nurses need a better support in this particular field (Baginska et al. 2019).

Almost 80% of respondents declared that their knowledge of caries prevention was at a sufficient level, which is similar to the results presented by Skeie et al. (2011) with regard to Norwegian public health nurses. In their study, nurses with longer work experience assessed themselves more positively, whereas the present survey showed opposite results. It is obvious that non-dental staff members need to acquire the knowledge to become competent and skilled in providing the oral health education (Tetuan et al. 2005; Dolce et al. 2012). Golinveaux et al. (2013) proved that an interdisciplinary educational intervention was sufficient for an improvement in the paediatric nurse practitioners’ knowledge of oral health topics, confidence when providing oral health counselling and attitudes about including oral health in their examinations. However, Polish nurses only occasionally received an institutional support in this field—less than a half of them declared the participation in any training on caries prevention. The course programme, currently applicable in Poland, preparing nurses to work in an education environment comprises issues concerning the oral disease prevention. However, most respondents had very long relevant work experience and probably their—mostly secondary—education did not comprise such issues. The fact that dental topics are missing during nurse’s education was previously reported in the literature. Skeie et al. (2011) reported that dental issues were a minor part of the educational curriculum for Norwegian public health nurses and in some institutions the subject was totally absent. On the other hand, Dolce et al. (2018) found that in USA the majority of programmes educating nurse practitioners included an oral health curriculum. The students’ satisfaction was greater when dental professionals or non-dental oral health experts were involved in the teaching process.

Undoubtedly dentists should support school nurses in their oral health education actions on a permanent basis. This situation may improve as a result of the Act on Pupils’ Healthcare (Polish Ministry of Health 2019) which was passed in 2019 in Poland. It stipulates undertaking, as part of healthcare integration, joint actions by school nurses, dentists and dental hygienists in the field of disease prevention, promotion of health and health education as well as identification of oral health risk factors and hazards. Further research is necessary to assess whether this policy will change school nurses’ attitudes and practices with regard to the oral health education. Due to national guidelines, Norwegian health nurses collaborate with dentists and, according to Løken et al. (2016), it is satisfactory for health nurses because they may receive sufficient information to provide oral health promotion and to identify children at risk of developing caries. However, data from the literature indicate that interprofessional education is needed to improve nurse’s oral health capacity and to prepare nurses and dentists for the interdisciplinary team-based work (Cooper et al. 2017; Nash et al. 2018). The result of partnership between the New York University College of Nursing’s and the New York University College of Dentistry was the Oral Health Nursing Education and Practice programme launched to prepare nurses for oral health tasks. Its outcomes included the production of a short film about the role of school nurse in improving oral health, an initiation of an expert advisory committee, a development of national nursing action plan, starting an electronic newsletter and conducting a train-the-trainer workshop. The development of an open access website serving as the educational infrastructure was intended (Dolce et al. 2012).

A major limitation of this study is a low response rate, however, a similar response rate was reported in other studies on the role of nurses in oral health education and nutrition counselling (Skeie et al. 2011; Ilmonen et al. 2012). The lack of information about the non-respondents may cause some bias to obtained results.

## Conclusion

Polish school nurses have potentially crucial roles in improving the oral health status in children and adolescents through oral hygiene education, dietary counselling and fluoride prophylaxis included in their duties. Despite the lack of training on caries prevention methods and a low support from dentists, they feel confident about their knowledge. Dentists should support school nurses in their oral health promotion actions.

## Data Availability

The data that support the findings of this study are available from the corresponding author upon reasonable request.
